# Education Interventions to Improve Knowledge, Beliefs, Intentions and Practices with Respect to Dietary Supplements and Doping Substances: A Narrative Review

**DOI:** 10.3390/nu13113935

**Published:** 2021-11-03

**Authors:** Jana Daher, Dalia El Khoury, John J. M. Dwyer

**Affiliations:** Department of Family Relations and Applied Nutrition, University of Guelph, 50 Stone Road, Guelph, ON N1G 2W1, Canada; jdaher@uoguelph.ca (J.D.); dwyer@uoguelph.ca (J.J.M.D.)

**Keywords:** education interventions, knowledge, intention, practices, dietary supplements, doping substances, athletes, general population

## Abstract

The misuse of dietary supplements and doping substances is commonly associated with toxicity, nutritional imbalances, and health and psychological consequences. This is alarming especially in light of the increasing prevalence of the use of dietary supplements and doping, particularly among young adults including athletes. There is evidence that education interventions can lead to improved knowledge, intentions, and practices. However, no review has summarized and evaluated the effectiveness of such interventions. The aim of this article is to review the characteristics, contents and effects of education interventions that were designed and implemented to improve knowledge, attitudes, beliefs and intentions with respect to the use of dietary supplements and doping agents in different populations. PubMed, Scopus, CINAHL, PsycInfo and Google Scholar were searched for English-language education interventions targeting dietary supplements and doping substances. A total of 20 articles were identified and have generally provided consistent findings. Most interventions reported a significant improvement in knowledge on dietary supplements and doping agents. Unfortunately, the heavy reliance on self-reported assessment tools limits the validity of these interventions, with almost all articles targeting athletes and adolescents.

## 1. Introduction

Natural health products, commonly known as dietary supplements, are naturally occurring substances used for the purpose of restoring or maintaining good health [1]. These include proteins, ergogenic supplements, vitamins, minerals, herbs and botanicals [2]. Generally, dietary supplements are commercially available and sold over the counter as tablets, capsules, gummies, powders, drinks and energy bars [3]. Until now, there has been no consensus on a clear definition and consistent categorization of dietary supplements [4,5], which can complicate the attempts to provide an overview of the current state of knowledge and pose multiple challenges to the interpretation of relevant research [2].

Dietary supplementation is generally needed for people following a low-energy diet, eliminating at least one food group from their diets, using severe weight-loss practices or consuming a high-carbohydrate diet poor in vitamins and minerals [6]. However, the use of dietary supplements is becoming increasingly prevalent even in populations whose diets are not deficient in nutrients [7], making it a multi-billion-dollar industry [8]. Athletes and physically active individuals represent a major part of dietary supplements users [9], for reasons including but not limited to improving physical performance [10], enhancing the rate of exercise recovery [11], health maintenance and increasing energy [12], and correcting nutritional deficiencies [13]. Dietary supplement usage by adolescents [14] and non-athlete university students [12] has been on the rise as well and is expected to continue to grow.

The indiscriminate use of supplements is problematic since a large body of evidence has shown that supplement users still rely on unreliable sources of information with regard to supplementation [15] such as family and friends, teammates, coaches, the Internet or their own judgement [12]. Moreover, dietary supplement misuse can expose users to harmful substances or precursors of prohibited substances [16,17], potentially leading to adverse health effects [18]. In light of the emerging concerns regarding their safety, several studies have investigated dietary supplements’ integrity and authenticity. These reported the presence of toxic element contamination [19], prohibited stimulants and anabolic androgenic steroids [20,21,22], and active pharmaceuticals, which can lead to serious health effects [23]. Contamination can occur either due to inadequate manufacturing procedures or can be intentional by manufacturers to increase the effectiveness of supplements [21]. The most frequently reported undeclared contaminants of dietary supplements are anabolic androgenic steroids and stimulants [22], which are mostly found in supplements used for enhancing athletic performance [21]. Current data have reported that consumers are unaware of the harmful potential of dietary supplements [15,24] and have unintentionally consumed supplements contaminated with anabolic steroids, prohormones, selective androgen receptor modulators (SARMs) and aromatase inhibitors that were not mentioned on the label [21,25,26]. However, some athletes intentionally use illegal doping agents, mainly androgenic anabolic steroids, to improve their physical performances in sports. Even among adolescents, the intentions to use doping substances are also affected by muscularity concerns, especially among boys [27]. Doping substance abuse is associated with serious health risks, especially when consumed in supra-physiological doses [28] and can lead to psychological side effects such as aggression, violent behavior, mood swings and mania [29].

Interestingly, it has been proposed that dietary supplements can be a gateway to doping and that the use of legal performance-enhancing dietary supplements can increase the probability of future doping substance use. Several studies have found a relationship between supplement use and doping susceptibility, which is defined as the absence of a solid decision to not engage in doping [30,31,32]. It was suggested that the routine use of dietary supplements in the sporting context can increase the users’ tendency to use doping agents based on their common intended outcome of maximizing performance [32].

With that being said, education interventions that target susceptible groups such as adolescents and athletes are indispensable for a greater awareness concerning dietary supplements and doping agents. It is important to find out the best approach to design education interventions targeting dietary supplements and doping substances, and this is not possible without studying the existing literature and identifying limitations and gaps. To our knowledge, there is no review in the literature that has evaluated education interventions targeting dietary supplements and doping agents and their effectiveness in improving knowledge, attitudes, intentions, and practices regarding supplement use.

The aim of this article is to review education interventions that were designed to improve knowledge, intentions, and practices regarding dietary supplements and doping agents in different populations, with a focus on at-risk ones including athletes and adolescents. Although this review presents an exhaustive search of the available literature, it is narrative in nature.

## 2. Literature Search

Searches were conducted on the electronic databases of PubMed, Scopus, CINAHL, PsycInfo, and Google Scholar for studies published up until July 2021. The search was restricted to English-language trials, but studies were eligible for inclusion regardless of the country they took place in. The following subject headings or keywords were used in the search: “doping”, “supplement”, “performance-enhancing”, “steroids”, “education”, “program”, “intervention”, “workshop”, “seminar”, and “campaign”. Keywords were combined through advanced search strings to find relevant articles. Reference lists of all retrieved articles were hand-searched for additional relevant studies.

## 3. Interventions

A full description of the reviewed interventions is detailed in Table 1

### 3.1. Intervention Demographics

Twenty-five studies were included in this review. The interventions were carried out in a diverse range of countries. Seven studies were conducted in the United States of America [41,42,43,44,48,54,56], five in the United Kingdom [45,47,50,51,53], three in Greece [35,47,53], two in Iran [33,46], two in Italy [37,49], one in Japan [36], Spain [34], Sweden [52], Germany [39], Malaysia [40], Australia [57], Canada [38], and Norway [55]. The sample size ranged from 35 to 20,800 and participants were older than 12 years of age.

The majority of the interventions (16 out of 22) involved adolescents [33,34,35,37,48,49,52,55] and young athletes [38,44,45,47,51,54,56,57].

### 3.2. Intervention Charactersitics

#### 3.2.1. Intervention Procedures and Modes of Delivery

The majority of the studies used multiple procedures to deliver their curriculum contents. The most used were lectures and presentations [36,37,40,42,43,48,51,52,55,58], group discussions [33,34,40,46,47,48], and written materials in the form of booklets, handouts, leaflets, posters, brochures and pamphlets that were mainly used in studies that used active control groups [40,41,42,43,44]. Some interventions used role playing [33,47] and physical activity including weight room training sessions and strength training sessions [43,44,45,46,47,48,49,50,51,52,53,54,55]. Others also used online presentations [45,51], problem-solving [37,47], and seminars [37,49].

All studies comprised a face-to-face format except for three [39,45,51]. It is surprising that for studies which were conducted online only, there was either no intervention effect [39], a significant increase in doping likelihood attitude scores [39], or a return of improved outcome measures to baseline scores at follow-up [45]. This was the case of the study of Elbe and Brand (2016) in which they reported attenuated doping rejection in the ethical decision-making group after the intervention and no intervention effect in the standard-knowledge-based educational program group [39]. In the study of Hurst et al. (2020), doping likelihood returned to baseline at 3-month follow-up [45]. It is also worth mentioning that in Nicholls et al.’s (2020) study [51], which used three delivery formats (face-to-face, online, hybrid), the doping susceptibility effects were only maintained in the face-to-face intervention group; however, attitudes towards doping were reduced and sustained at follow-up in all groups.

#### 3.2.2. Curriculum Content and Intervention Providers

The interventions covered comprehensive information on a range of topics, including but not limited to the safety of dietary supplements, quality of dietary supplements, possibility of interactions between dietary supplements and medicines, sport nutrition, substances and methods of doping, anti-doping rule violations, side effects of androgenic anabolic steroids use, risks associated with sports supplements, and general information about the World Anti-Doping Agency. Trials that had an ethical and moral aspect have focused on dilemmas related to doping, famous doping cases, law and punishment, the role that media messages can have in dysfunctional beliefs, and the moral and ethical implications of doping and substance use in their intervention contents [39].

The interventions were delivered by the investigators themselves [36,40,48], trained facilitators/staff [33,44,47,53,55], physical education teachers [34,35,57], coaches and squad leaders [41,54], track and field athletes [45], and health workers [52]. Some studies were implemented by a combination of different professionals to tackle different aspects of the intervention. For example, the intervention in Lucidi et al.’s (2017) study was provided by communication experts, pharmacology experts, high-level sport athletes, and sport psychologists [49], while that of Codella et al. (2019) was delivered by track and field coaches, sport scientists, sport psychologists and physicians [37]. It is important to note that none of the interventions were delivered by trained educators, despite the fact that most of them are educational in nature. Therefore, although the intervention providers might be very knowledgeable about the intervention topic, they might not be experts in communicating information and delivering it to the target audience.

#### 3.2.3. Use of Theory

An added value of evaluating theory-guided interventions is that a specified intervention’s outcomes and the change in the theoretical constructs can be measured [58]. Theory-based interventions provide an opportunity to discern which components work and which do not [59]. In this review, several studies specified the use of a behavioral change theory [46,49,55]. Behavioral change theories back up interventions through explaining how behaviors change and describing what factors influence them [60]. Jalilian et al. (2011) implemented the theory of planned behavior (TPB) [46], first introduced by Ajzen (1985) [61], which is one of the theories used to predict and understand behavior. The theory postulates that attitude, subjective norms, and perceived behavioral control shape behavioral intentions [61]. Despite the use of the TPB theoretical framework, the findings showed that the education program did not improve subjective norms and perceived behavioral control against anabolic androgenic steroids but increased behavioral intentions to not use them. Another study by Lucidi et al. (2017) [49] based their intervention on the cognitive theory of media literacy, which aims to give a person a greater control of exposure to media messages and a greater awareness of the implications of these messages [62]. The authors claimed that it is the first research work to show the efficacy of media literacy concerning “performance and appearance enhancing substances” in adolescents [49]. Sagoe et al. (2016) [45] designed their program based on the Social Learning Theory, which proposes that behaviors are acquired through the observation of others [63]. They also used the health belief model, which suggests that actions related to drug use depend on the perceived susceptibility to and the severity of drug effects [64]. The program was also anchored in the TPB. When combined with practical strength training, theoretical lessons were better at improving knowledge on anabolic androgenic steroids and awareness of their negative consequences. The Adolescents Training and Learning to Avoid Steroids Program (ATLAS), which was implemented in a number of studies [43,44,57], was also based on the Social Learning Theory [63].

## 4. Research Design

Table 2 presents the research designs and key findings of the reviewed interventions grouped by design quality from highest to lowest. The majority of the interventions were delivered using a pretest post-test control group design [35,38,41,42,43,44,46,47,50,51,53,54,55,56], which controls for several threats to internal validity [65]. However, other studies used quasi-experimental designs [33,34,39,40,48,49,57], which does not guarantee that the groups were comparable at baseline. Four studies used a pre-experimental design [36,37,45,52], which is associated with multiple threats to internal validity, such as history, maturation, testing, and statistical regression [65]. This research design does not control for factors that might have caused the change after the intervention.

## 5. Outcome Measures and Key Findings

Each of the trials included in the review assessed a variety of outcomes. Knowledge, attitudes, intentions, and use were the most commonly reported. The majority of the outcome measures were self-reported by participants in the interventions. Various assessment methods were used but the most common were self-completed questionnaires.

### 5.1. Knowledge

Most of the interventions assessed changes in knowledge. Chiba et al. (2020) assessed the students’ understanding of dietary supplements using a questionnaire, which revealed that students’ understanding of dietary supplements improved among users and non-users post intervention [36]. This is consistent with the findings of another study in which sports nutrition knowledge increased among team sports athletes [40]. Little et al. (2002) also assessed nutrition supplement knowledge among high school students from a low-income community. They found a significant improvement in knowledge in the experimental group, especially among females [48]. This is consistent with the findings of another study in which knowledge about anti-doping rules improved after a 60-min session [45]. It is interesting to learn about short, yet effective education interventions. However, Álvarez Medina et al.’s (2019) study, in which a school-based doping prevention program improved knowledge about doping, indicated that some information requires more time to be assimilated, which questions the effectiveness of short interventions [34]. Goldberg et al. (1990) reported increased knowledge on the effects of anabolic steroids after an education program [42]. This is consistent with the results of Jalilian et al.’s (2011) study, in which an anabolic steroid preventative intervention improved knowledge about the side effects of androgenic anabolic steroids [46]. Interestingly, combining practical strength training with theoretical lessons was found to be better at increasing knowledge on anabolic androgenic steroids than theory alone [55]. In general, all the studies were reported to be effective in improving knowledge on dietary supplements and doping substances. It is difficult to compare interventions due to the use of different designs, assessment methods, modes of delivery, procedures, and intervention durations.

### 5.2. Attitude and Intention

The attitudes and intentions of participants towards using doping agents improved in most of the interventions [33,34,35,40,44,45,46,49,51,54]. Improved doping attitudes and intentions were maintained for 8 weeks in one study [51] and for 9 months in another [54], but returned to baseline in the intervention group in Hurst et al.’s (2020) study at 3-month follow-up [45]. However, there were no differences in attitudes toward anabolic steroids use in the study by Goldberg et al. (1990) post intervention [42]. The authors claimed that this might be due to a greater familiarity with the questionnaire among the participants since the same questionnaire was used before the intervention [42]. It is surprising that doping attitudes increased in the ethical decision-making training group in the study by Elbe et al. (2016) [39]. In this study, participants were assigned to an ethical decision-making group that comprised six sessions with three dilemmas each that dealt with fictious young athletes, or a standard-knowledge-based education program group that also entailed six education sessions on doping. The researchers explained that this can be an indication that the ethical decision-making training succeeded in breaking up the stereotypical style of reasoning about doping among athletes. Although most studies were effective in improving attitudes and intentions towards doping, some did not succeed at improving or maintaining them [39,42]. Some researchers have argued that drug education can produce effects counter to those intended, similar to the case of increased attitudes towards doping [66,67]. While all the studies assessed knowledge, only part of them assessed attitudes and intentions; therefore, there should be more focus on attitudes and intentions to be able to make inferences on whether there is any unintended boomerang effect of education interventions.

### 5.3. Use

The use of supplements or doping substances was assessed in a few interventions only and was mainly self-reported. Jalilian et al. (2011) reported a decreased rate of anabolic androgenic steroid and dietary supplement use in the intervention group, but this decrease was not statistically significant [46]. This was attributed to the low sample size, limitation of resources to design a comprehensive education program, and peer pressure. Lucidi et al. (2017) [49] reported a statistically significant decrease in self-reported doping use in their intervention group despite the fact that the intervention was six months shorter than that of Jalilian et al. (2011) [46]. Consistently, Nilsson et al. (2004) reported decrements in androgenic anabolic steroids use after a 2-year intervention focusing on raising self-confidence and awareness on appearance ideals and providing information on androgenic anabolic steroids [52]. While all studies reported decrements in supplement and doping use, the use of self-reported questionnaires increases the risk of social desirability bias, which tends to increase with a sensitive topic such as doping [68]. Nonetheless, self-reported measures might be the only ethical option given the possible implications of other methods (i.e., being banned).

## 6. Gaps in the Literature and Limitations

There is a lack of education interventions addressing dietary supplements and doping substances in many countries and for different age groups. Current interventions mainly focus on adolescents and athletes, with very few implementing interventions among non-athlete adults and older adults. Sex-specific education interventions should also be considered in future studies since females and males may respond differently to education interventions [39]. There is also a need for these interventions to be based on behavior change theories to better understand the main determinants of reported behavioral changes [58,59]. Moreover, most of the current literature uses self-reported questionnaires to assess the effects of education interventions, which is more likely to be biased due to social desirability. There is also a limited number of technology-based interventions, most of which did not have effects on the participants. Therefore, further education interventions that are designed using an adequate behaviour change theory as a framework, use valid and reliable questionnaires, and incorporate technology-based tools need to be designed and implemented to address these limitations.

## 7. Conclusions

There is an increasing interest in understanding the value of education interventions in improving knowledge, beliefs, intentions, and practices regarding the use of dietary supplements and doping substances, targeting specifically those at risk such as young adults including athletes. The findings from this review confirm that such interventions have promising results, especially in improving knowledge. However, the heavy reliance on self-reported outcome measures limits the validity of these results.

## Figures and Tables

**Table 1:** Study demographics and intervention characteristics.

Study Demographics				Intervention Characteristics			
**Publication-Country**	Target Population	Age Mean ± SD (Years)	Sex (Sample Size)	Intervention Procedure/Mode of Delivery	Intervention Duration	Intervention Provider	Curriculum
Allahverdipour et al. (2009) Iran [33]	Urban Iranian high school students	EG: 16.1 ± 0.8 CG: 16.2 ± 0.6	M (n = 189)	EG: Role playing, group discussions. (Face to face.) CG: Passive controls	3 months. (60-min sessions, twice a week.)	Trained study staff	EG: Side effects and consequences of substance use.
Álvarez et al. (2019) Spain [34]	Adolescents	12–13	M (n = 270) F (n = 270)	EG: Physical activity, group debate. (Face to face.) CG: Passive controls.	One month. (Six 55-min sessions, twice a week.)	Physical education teachers	EG: Doping, principles of fair play, substances and methods of doping, importance of struggle against doping, the values of the true “spirit of sport”.
Barkoukis et al. (2016) Greece [35]	High school students	EG: 16.1 ± 1.7 CG: 15.8 ± 0.7	M (n = 107) F (n = 110)	Classroom sessions. (Face to face.)	20 h. (1.5 h per week.)	Physical education teachers	EG: Health, moral, social, and psychological aspects of nutritional supplement and doping use. CG: Other health-related issues (recycling, bullying, psychological well-being)
Chiba et al. (2020) Japan [36]	College students	18–38 years	M (n = 150) F (n = 178)	Lecture-based. (Face to face.)	7 months. (1-h lecture.)	First author	Safety of dietary supplement use, quality of DS, the possibility of interactions between DS and medicines, adverse events that might occur due to their use.
Codella et al. (2019) Italy [37]	High school students	15–18 years	(n = 20,800)	Seminar (PowerPoint presentation, scenario-analysis, problem solving, group discussion). (Face to face.)	2 h.	Expert leaders (track and field coaches, sport scientists, sport psychologists, physicians)	Doping-related medical aspects, psychological aspects, and athletic coaching.
Duncan and Hallward (2019) Canada [38]	Adolescent athletes	13.7 ± 1.3	M (n = 70) F (n = 63)	Gain-framed or loss-framed messages. (Video.)	5-min video.	Research team	Physical, psychological, social, and moral reasons to avoid doping.
Elbe and Brand (2016) Germany [39]	Young elite athletes.	15.5 ± 2.4	M (n = 34) F (n = 35)	Educational sessions. (Online.) CG: Passive controls.	2 weeks. (Six 30-min online sessions.)	Research team	Ethical decision-making group: Dilemmas related to doping. Standard knowledge-based educational program: Precursors and consequences of doping, forbidden substances, doping control system, law and punishment, and doping on the internet.
Elias et al. (2018) Malaysia [40]	Malaysian team sports athletes	EG: 18.7 ± 0.9 CG: 23.3 ± 3.8	M (n = 105)	EG: Educational booklets, lecture sessions, group discussions, and group activities. (Face to face.) CG: Passive controls.	7 weeks. (1–2 h per week.)	Researchers	Food and healthy nutrition, macro and micronutrient, fluid and hydration, nutrition before, during and after training, energy balance and weight management, dietary supplements.
Elliot et al. (2004) USA [41]	High school athletes	EG: 15.4 ± 1.2 CG: 15.4 ± 1.2	F (n = 928)	EG: Classroom sessions. (Face to face.) CG: Pamphlets	8 weeks. (Eight 45-min classroom sessions.)	Coaches and squad leaders	EG: Healthy sport nutrition, effective exercise training, drug use, unhealthy behaviors’ effects on sport performance, media images of females, depression prevention. CG: Disordered eating, drug use, and sports nutrition.
Goldberg et al. (1990) USA [42]	Varsity high school football teams	17 ± 1	(n = 190)	EG1: Oral presentation, Q/A session, handouts. (Face to face.) EG2: Education handout. CG: Passive controls.	20-min presentation.	NR	Information on The American College of Sports Medicine’s (ACSM) position on the use of anabolic androgenic steroids in sports.
Goldberg et al. (1996) USA [43]	High school football teams	EG: 15.9 ± 1.1 CG: 15.7 ± 1.1	M (n = 1506)	EG: classroom sessions, weight-training sessions. (Face to face.) CG: Pamphlet.	7 weeks. (Seven 50-min class sessions and 7 weight-training sessions.)	Coaching staff, peer educators and staff trainers.	Anabolic androgenic steroids (AAS) effects, sports nutrition, strength-training alternatives to AAS use, drug refusal role play, and anti-AAS media messages. CG: Problems associated with AAS use, ethics of fair play and sportsmanship.
Goldberg et al. (2000) USA [44]	High school football teams	EG: 15.5 ± 1.2 CG: 15.4 ± 1.2	M (n = 3207)	EG: Classroom and exercise training sessions. (Face to face.) CG: Pamphlet.	Cohort 1: Seven 45-min classroom and seven weight-room sessions. Cohorts 2 and 3: Five 45-min- classroom sessions and three weight room sessions.	Coaching staff, peer facilitators and trainers.	EG: Sports nutrition, exercise alternatives to anabolic steroids and sport supplements, effects of substance abuse in sports, drug-refusal role playing, and the creation of health promotion messages. CG: Adverse effects of anabolic steroids and benefits of a sports nutrition diet.
Hurst et al. (2020) UK [45]	Junior elite athletes	17.2 ± 0.7	(n = 202)	Electronic presentation in a classroom setting. (Online.)	60-min session.	Track and field athlete	Information about the World Anti-Doping Agency, drug testing, anti-doping rule violations, use of medications, and risks associated with sport supplements.
Jalilian et al. (2011) Iran [46]	Young gym users	EG: 24.4 ± 5.5 CG: 23.0 ± 3.1	M (n = 120)	EG: Group discussion, printed leaflet, and audio-visual CD. (Hybrid.) CG: Passive controls	Six 1-h sessions.	Mediator	EG: Side effects of anabolic androgenic steroids (AAS) abuse.
Kavussanu et al. (2020) UK and Greece [47]	Athletes	16–20	M (n = 201) F (n = 102)	Video, group discussion, role -play, problem solving. (Hybrid.)	Six 1-h sessions.	Trained facilitator	- Moral intervention: success in sport, values in sport, justifications for doping, consequences of doping for others, the culture of the team. - Education intervention: doping control, banned substances, sport supplements, nutrition, whistleblowing.
Little et al. (2002). USA [48]	Adolescents	NR	M (n = 15) F (n = 24)	EG: Lectures, group activities, class discussion. (Face to face.) CG: Passive controls.	Seven weeks. (Five 1-h sessions.)	Investigators	EG: Vitamins, minerals, water, protein supplements, creatine monohydrate, fat-burning supplements, steroid-alternative supplements, muscle-sparing supplements, sports drinks.
Lucidi et al. (2017) Italy [49]	High school students	EG: 16.6 ± 1.3 CG: 16.1 ± 1.4	(n = 492)	EG: Seminars, meetings. (Face to face.) CG: Regular school classes.	Six months. (Twelve 90-min sessions/twice a month.)	Communication experts, pharmacology experts, high-level sport athletes, sport psychologists	EG: Role that media messages can have in promoting dysfunctional beliefs, side effects of doping substances, moral and ethical implications of doping substance use, beliefs and strategies in re-framing awareness and sport-related goals. CG: Physical education or health education classes.
Nicholls et al. (2020) UK [50]	Coaches	EG: 38.9 ± 11.9 CG: 42 ± 14	M (n = 154) F (n = 33)	EG: Mobile application. CG: Passive controls.	Six weeks.	Mobile application	CG: Fair play, substances, nutritional supplements, rules, and leadership.
Nicholls et al. (2020) UK [51]	High-level adolescent athletes	CG: 15.9 ± 1.6 Presentation: 16.5 ± 1.1 Online: 15.9 ± 1.3 Presentation + online: 16.2 ± 1.3	M (n = 904) F (n = 177)	EG: Presentations (3 groups). (Face-to-face presentation, online access, and blended face-to-face and online access.) CG: Normal training.	Face to face: two 90-min presentations, 8 weeks apart. Online: online access Face to face and online: two 90-min presentations, 8 weeks apart, then online access.	NR	Introduction to doping, goals, motivation, doping myths, playing fair, resisting temptations, making the right decisions, drug testing and health, nutritional supplements, and coping strategies.
Nilsson et al. (2004) Sweden [52]	Adolescents	16–17 years	(n = 921)	Teaching sessions, group discussions, posters, brochures, trailers. (Face to face)	Two years. (Number/duration of lectures NR.)	Health workers	Androgenic anabolic steroids.
Ntoumanis et al. (2021) Australia, UK, Greece [53]	Coaches and athletes.	EG: - Athletes: 22.4 ± 11.4 - Coaches: 39.7 ± 14.6 CG: - Athletes: 18.6 ± 7 - Coaches: 36.8 ± 12.1	(n = 130)	EG: Motivationally enriched anti-doping education workshops. (Face to face.) CG: Standard anti-doping education workshop. (Face to face.)	12 weeks (EG: Two 3-h workshops.) CG: 1-h workshop.)	Trained facilitators	EG: Doping prevention + supportive communication (training coaches on applying supportive communication to discuss doping-related issues with their athletes). CG: Doping prevention
Ranby et al. (2009) USA [54]	High school female athletes	NR	F (n = 1668)	EG: Facilitated sessions. (Face to face.) CG: Pamphlets.	8 weeks (eight weekly 45-min sessions).	Coach	EG: Sports nutrition, body shaping substances, supplements and diet pills. CG: Disordered eating, drug use, sports nutrition.
Sagoe et al. (2016) Norway [55]	High school students	EG1: 16.9 ± 0.4 EG2: 16.9 ± 0.7 CG: 16.6 ± 0.8	M (n = 104) F (n = 98)	EG1: Theoretical/classroom education + supervised strength training exercises. (Face to face.) EG2: Theoretical/classroom education. (Face to face.) CG: Passive controls.	12 weeks (Theoretical/classroom education: four 90-min sessions Training: 12 sessions.)	Staff of Anti-doping Norway	Theoretical/classroom education: Basic and biomechanical principles of exercise and strength training, nutrition and dietary supplementation, sports ethics, anti-doping, anabolic steroids and their adverse health effects, dealing with peer pressure. Practical training: squat, deadlift, bench press, standing shoulder press, lat pulldown, seated row, and standing barbell twist.
Trenhaile et al. (1998) USA [56]	Preadolescents	9–12 years	M (n = 35)	EG: Sessions. (Face to face.) CG: Passive controls.	Two weeks (six 30-min lessons).	Teacher	EG: Psychological and physiological aspects of anabolic steroid use, weight training techniques, nutrition, social decision-making, and self-esteem training.
Yager and McLean (2019) Australia [57]	Grade 10 boys	EG: 15.9 ± 0.4 CG: 15.7 ± 10.3	M (n = 211)	EG: Classroom sessions. CG: Passive controls.	5 weeks (ten 45-min sessions).	Physical education teachers	EG: Drug and supplement education, strength training, sports nutrition.

Notes: (EG), experimental group; (CG), control group; (F), female; (M), male; (NR), not reported. The majority of the participants resided in the UK.

**Table 2:** Research Designs and Key Findings.

Research Design	Study	Notation	Key Findings
**True Experimental**	Barkoukis et al. (2016) [35]	R O_1_ X_1_ O_2_ R O_1_ X_2_ O_2_	Significantly weaker attitudes towards doping use and increased norm salience in EG.
	Duncan and Hallward (2019) [38]	R O_1_ X_1_ O_2_ R O_1_ X_2_ O_2_	No differential influence for either message frame on changes in any of the outcomes. Attitudes, self-efficacy, and perceived norms all increased significantly over time for participants in both conditions.
	Elliot et al. (2004) [41]	R O_1_ X_1_ O_2_ R O_1_ X_2_ O_2_	Significantly lower use of diet pills and athletic-enhancing substances in EG, and reduced intentions toward future use of diet pills and other health-harming actions.
	Goldberg et al. (1990) [42]	R O_1_ X_1_ O_2_ R O_1_ X_2_ O_2_ R O_1_ O_2_	Increased awareness of adverse effects of anabolic steroids in EG, but no differences in attitudes toward its use.
	Goldberg et al. (1996) [43]	R O_1_ X_1_ O_2_ R O_1_ O_2_	Increased understanding on anabolic androgenic steroids (AAS), greater belief in personal vulnerability to the adverse consequences of AAS, improved drug refusal skills, less belief in AAS-promoting media messages and reduced intentions to use AAS in the EG.
	Goldberg et al. (2000) [44]	R O_1_ X_1_ O_2_ R O_1_ O_2_	Lower intentions to use and actual use of anabolic steroids in EG. Reduced illicit drug use, drinking and driving, and sport supplement use in favor of EG along with improved nutrition behaviors.
	Jalilian et al. (2011) [46]	R O_1_ X_1_ O_2_ R O_1_ O_2_	Significant improvements in knowledge about side effects of AAS, attitude toward, and intention not to use AAS. Decrements in the rate of AAS and supplements use in the EG.
	Kavussanu et al. (2020) [47]	R O_1_ X_1_ O_2_ O_3_ O_4_ R O_1_ X_2_ O_2_ O_3_ O_4_	Lower doping likelihood and moral disengagement and higher guilt from pre to post intervention in both groups. Effects maintained at 3 and 6 months follow-up in both groups as well.
	Nicholls et al. (2020) [50]	R O_1_ X_1_ O_2_ R O_1_ O_2_	Increased knowledge about doping and reduced favorable doping attitudes in the EG.
	Nicholls et al. (2020) [51]	R O_1_ X_1_ O_2_ R O_1_ X_2_ O_2_ R O_1_ X_3_ O_2_ R O_1_ O_2_	Reduced favorable attitudes towards doping and sustained for 8 weeks in all intervention groups compared to CG. Doping susceptibility effects were only maintained for face-to-face presentation group.
	Ntoumanis et al. (2021) [53]	R O_1_ X_1_ O_2_ R O_1_ X_2_ O_2_	Athletes in EG reported greater reductions in willingness to take prohibited substances post intervention, but not at follow-up. Coaches in the EG reported greater increases in efficacy to create an anti-doping culture.
	Ranby et al. (2009) [54]	R O_1_ X_1_ O_2_ O_3_ R O_1_ X_2_ O_2_ O_3_	Decreased intentions for steroid/creatine use and intentions for unhealthy weight loss behaviors in EG. Low intentions were maintained 9 months later in EG.
	Sagoe et al. (2016) [55]	R O_1_ X_1_ O_2 _ R O_1_ X_2_ O_2 _ R O_1_ O_2_	Higher knowledge of AAS and their harmful effects as well as a higher increase in strength training self-efficacy in “theory with workout group (EG1)”.
	Trenhaile et al. (1998) [56]	R O_1_ X_1_ O_2 _ R O_1_ O_2_	Improved knowledge of anabolic steroids and stronger attitudes against using steroids in the future in EG.
**Quasi-Experimental**	Allahverdipour et al. (2009) [33]	O_1_ X O_2_ O_1_ O_2_	Significantly improved substance knowledge, attitudes, peer resistance skills, level of self-control, self-efficacy, and perceived susceptibility among EG. Deteriorated level of self-control and attitudes against substance abuse among CG.
	Álvarez et al. (2019) [34]	O_1_ X O_2_ O_1_ O_2_	Improved knowledge, attitudes, and beliefs about doping in favor of EG.
	Elbe and Brand (2016) [39]	R O_1_ X_1_ O_2_ R O_1_ X_2_ O_2_ O_1_ O_2_	Increased doping likelihood attitudes (attenuated doping rejection) in the ethical decision-making group. No intervention effect in both the standard-knowledge-based educational program group and the control group.
	Elias et al. (2018) [40]	O_1_ X O_2_ O_1_ O_2_	Increments in mean scores of sports nutrition knowledge and practice in EG compared to decrements in respective scores in CG. Improved dietary intake in favor of the EG.
	Little et al. (2002) [48]	O_1_ X_1_ O_2_ O_1_ X_2_ O_2_	Improved nutrition and sport supplement knowledge in EG.
	Lucidi et al. (2017) [49]	O_1_ X_1_ O_2_ O_1_ O_2_	Stronger attitudes against doping use, decreased self-reported supplement use in EG.
	Yager and McLean (2019) [57]	O_1_ X_1_ O_2_ O_1_ O_2_	Improved body satisfaction and increased negative attitudes toward substance and supplement use in EG; however, these changes were not significant after adjusting for multiple comparisons.
**Pre-Experimental**	Chiba et al. (2020) [36]	O_1_ X O_2_	Significantly improved understanding of dietary supplements.
	Codella et al. (2019) [37]	O_1_ X O_2_	Increased level of knowledge about anti-doping rules, legitimacy, and nutrition supplements.
	Hurst et al. (2020) [45]	O_1_ X O_2_ O_3_	More knowledge about anti-doping rules. Lower scores for intention to use supplements, beliefs about the effectiveness of supplements, doping likelihood, and doping moral disengagement. At follow-up, doping likelihood and moral disengagement returned to baseline.
	Nilsson et al. (2004) [52]	O_1_ X O_2_	Decreased misuse of anabolic steroids.

Notes: (X), intervention; (O), observation; (R), randomization. The subscripts 1 and 2 in this notation refer to the sequential order of the observations.

## References

[1] Health Canada Natural and Non-Prescription Health Products Directorate. https://www.canada.ca/en/health-canada/corporate/about-health-canada/branches-agencies/healthproducts-food-branch/natural-non-prescription-health-products-directorate.html.

[2] Garthe I., Maughan R.J. (2018). Athletes and Supplements: Prevalence and Perspectives. Int. J. Sport Nutr. Exerc. Metab..

[3] National Institutes of Health Dietary Supplements: What You Need to Know. https://ods.od.nih.gov/factsheets/WYNTK-Consumer/.

[4] Knapik J.J., Steelman R.A., Hoedebecke S.S., Austin K.G., Farina E.K., Lieberman H.R. (2015). Prevalence of Dietary Supplement Use by Athletes: Systematic Review and Meta-Analysis. Sports Med..

[5] Roy K.-A., El Khoury D., Dwyer J.J.M., Mountjoy M. (2020). Dietary Supplementation Practices among Varsity Athletes at a Canadian University. J. Diet. Suppl..

[6] Thomas D.T., Erdman K.A., Burke L.M. (2016). Position of the Academy of Nutrition and Dietetics, Dietitians of Canada, and the American College of Sports Medicine: Nutrition and Athletic Performance. J. Acad. Nutr. Diet..

[7] Oliver A.J.S., Leon M.T.M., Hernández E.G. (2008). Statistical analysis of the consumption of nutritional and dietary supplements in gyms. Arch. Latinoam. Nutr..

[8] Statista Total U.S. Dietary Supplements Market Size from 2016 to 2024. https://www.statista.com/statistics/828481/total-dietary-supplements-market-size-in-the-us/.

[9] Maughan R.J., King D.S., Lea T. (2004). Dietary supplements. J. Sports Sci..

[10] De Silva A., Samarasinghe Y., Senanayake D., Lanerolle P. (2010). Dietary Supplement Intake in National-Level Sri Lankan Athletes. Int. J. Sport Nutr. Exerc. Metab..

[11] Wiens K., Erdman K.A., Stadnyk M., Parnell J.A. (2014). Dietary Supplement Usage, Motivation, and Education in Young Canadian Athletes. Int. J. Sport Nutr. Exerc. Metab..

[12] El Khoury D., Hansen J., Tabakos M., Spriet L.L., Brauer P. (2020). Dietary Supplement Use among Non-athlete Students at a Canadian University: A Pilot-Survey. Nutrients.

[13] Erdman K.A., Fung T.S., Reimer R.A. (2006). Influence of Performance Level on Dietary Supplementation in Elite Canadian Athletes. Med. Sci. Sports Exerc..

[14] Tiwari K. (2020). Supplement (mis)use in adolescents. Curr. Opin. Pediatr..

[15] Baltazar-Martins G., De Souza D.B., Aguilar-Navarro M., Muñoz-Guerra J., Plata M.D.M., Del Coso J. (2019). Prevalence and patterns of dietary supplement use in elite Spanish athletes. J. Int. Soc. Sports Nutr..

[16] Parnell J.A., Wiens K., Erdman K.A. (2015). Evaluation of congruence among dietary supplement use and motivation for supplementation in young, Canadian athletes. J. Int. Soc. Sports Nutr..

[17] Schwenk T.L., Costley C.D. (2002). When Food Becomes a Drug: Nonanabolic Nutritional Supplement Use in Athletes. Am. J. Sports Med..

[18] Lazic J.S., Dikic N., Radivojevic N., Mazic S., Radovanovic D., Mitrovic N., Lazic M., Zivanic S., Suzic S. (2011). Dietary supplements and medications in elite sport—Polypharmacy or real need?. Scand. J. Med. Sci. Sports.

[19] Genuis S.J., Schwalfenberg G., Siy A.-K.J., Rodushkin I. (2012). Toxic Element Contamination of Natural Health Products and Pharmaceutical Preparations. PLoS ONE.

[20] Geyer H., Parr M.K., Koehler K., Mareck U., Schänzer W., Thevis M. (2008). Nutritional supplements cross-contaminated and faked with doping substances. J. Mass Spectrom..

[21] Mathews N.M. (2017). Prohibited Contaminants in Dietary Supplements. Sports Health A Multidiscip. Approach.

[22] Walpurgis K., Thomas A., Geyer H., Mareck U., Thevis M. (2020). Dietary Supplement and Food Contaminations and Their Implications for Doping Controls. Foods.

[23] Tucker J., Fischer T., Upjohn L., Mazzera D., Kumar M. (2018). Unapproved Pharmaceutical Ingredients Included in Dietary Supplements Associated with US Food and Drug Administration Warnings. JAMA Netw. Open.

[24] Tian H.H., Ong W.S., Tan C.L. (2009). Nutritional supplement use among university athletes in Singapore. Singap. Med. J..

[25] Martínez-Sanz J.M., Sospedra I., Ortiz C.M., Baladia E., Gil-Izquierdo A., Ortiz-Moncada R. (2017). Intended or Unintended Doping? A Review of the Presence of Doping Substances in Dietary Supplements Used in Sports. Nutrients.

[26] Tsarouhas K., Kioukia–Fougia N., Papalexis P., Tsatsakis A., Kouretas D., Bacopoulou F., Tsitsimpikou C. (2018). Use of nutritional supplements contaminated with banned doping substances by recreational adolescent athletes in Athens, Greece. Food Chem. Toxicol..

[27] Zelli A., Lucidi F., Mallia L. (2010). The relationships among adolescents’ drive for muscularity, drive for thinness, doping attitudes, and doping intentions. J. Clin. Sport Psychol.

[28] Birzniece V. (2015). Doping in sport: Effects, harm and misconceptions. Intern. Med. J..

[29] Kanayama G., Pope H.G. (2012). Illicit use of androgens and other hormones: Recent advances. Curr. Opin. Endocrinol..

[30] Backhouse S.H., Whitaker L., Petroczi A. (2011). Gateway to doping? Supplement use in the context of preferred competitive situations, doping attitude, beliefs, and norms. Scand. J. Med. Sci. Sports.

[31] Boardley I.D., Grix J., Harkin J. (2013). Doping in team and individual sports: A qualitative investigation of moral disengagement and associated processes. Qual. Res. Sport Exerc. Health.

[32] Hurst P., Kavussanu M., Boardley I., Ring C. (2019). Sport supplement use predicts doping attitudes and likelihood via sport supplement beliefs. J. Sports Sci..

[33] Allahverdipour H., Bazargan M., Farhadinasab A., Hidarnia A., Bashirian S. (2009). Effectiveness of skill-based substance abuse intervention among male adolescents in an Islamic country: Case of the Islamic Republic of Iran. J. Drug Educ..

[34] Lvarez J., Manonelles P., Grao-Cruces A., Oliete E., Murillo V., Nuviala A. (2019). Effectiveness of a school-based doping prevention programme in Spanish adolescents. J. Hum. Sport Exerc..

[35] Barkoukis V., Kartali K., Lazuras L., Tsorbatzoudis H. (2016). Evaluation of an anti-doping intervention for adolescents: Findings from a school-based study. Sport Manag. Rev..

[36] Chiba T., Kobayashi E., Okura T., Sekimoto M., Mizuno H., Saito M., Umegaki K. (2020). An educational intervention improved knowledge of dietary supplements in college students. BMC Public Health.

[37] Codella R., Glad B., Luzi L., La Torre A. (2019). An Italian Campaign to Promote Anti-doping Culture in High-School Students. Front. Psychol..

[38] Duncan L.R., Hallward L. (2019). An Experimental Test of the Efficacy of Gain- and Loss-Framed Messages for Doping Prevention in Adolescent Athletes. Subst. Use Misuse.

[39] Elbe A.-M., Brand R. (2014). The Effect of an Ethical Decision-Making Training on Young Athletes’ Attitudes Toward Doping. Ethics Behav..

[40] Elias S.S.M., Saad H.A., Taib M.N.M., Jamil Z. (2018). Effects of sports nutrition education intervention on sports nutrition knowledge, attitude and practice, and dietary intake of Malaysian team sports athletes. Malays. J. Nutr..

[41] Elliot D.L., Goldberg L., Moe E.L., DeFrancesco C.A., Durham M.B., Hix-Small H. (2004). Preventing substance use and disordered eating: Initial outcomes of the ATHENA program. Arch. Pediatr. Adolesc. Med..

[42] Goldberg L., Bosworth E.E., Bents R.T., Trevisan L. (1990). Effect of an anabolic steroid education program on knowledge and attitudes of high school football players. J. Adolesc. Health Care.

[43] Goldberg L., Elliot D., Clarke G.N., MacKinnon D.P., Moe E., Zoref L., Green C., Wolf S.L., Greffrath E., Miller D.J. (1996). Effects of a multidimensional anabolic steroid prevention intervention. The Adolescents Training and Learning to Avoid Steroids (ATLAS) Program. JAMA.

[44] Goldberg L., MacKinnon D.P., Elliot D.L., Moe E.L., Clarke G., Cheong J. (2000). The adolescents training and learning to avoid steroids program: Preventing drug use and promoting health behaviors. Arch. Pediatr. Adolesc. Med..

[45] Hurst P., Ring C., Kavussanu M. (2019). An evaluation of UK athletics’ clean sport programme in preventing doping in junior elite athletes. Perform. Enhanc. Health.

[46] Jalilian F., Allahverdipour H., Moeini B., Moghimbeigi A. (2011). Effectiveness of Anabolic Steroid Preventative Intervention among Gym Users: Applying Theory of Planned Behavior. Health Promot. Perspect..

[47] Kavussanu M., Hurst P., Yukhymenko-Lescroart M., Galanis E., King A., Hatzigeorgiadis A., Ring C. (2021). A Moral Intervention Reduces Doping Likelihood in British and Greek Athletes: Evidence from a Cluster Randomized Control Trial. J. Sport Exerc. Psychol..

[48] Little J.C., Perry D.R., Volpe S.L. (2002). Effect of nutrition supplement education on nutrition supplement knowledge among high school students from a low-income community. J. Community Health.

[49] Lucidi F., Mallia L., Alivernini F., Chirico A., Manganelli S., Galli F., Biasci V., Zelli A. (2017). The Effectiveness of a New School-Based Media Literacy Intervention on Adolescents’ Doping Attitudes and Supplements Use. Front. Psychol..

[50] Nicholls A.R., Fairs L.R.W., Plata-Andrés M., Bailey R., Cope E., Madigan D., Koenen K., Glibo I., Theodorou N.C., Laurent J.-F. (2020). Feasibility randomised controlled trial examining the effects of the Anti-Doping Values in Coach Education (ADVICE) mobile application on doping knowledge and attitudes towards doping among grassroots coaches. BMJ Open Sport Exerc. Med..

[51] Nicholls A.R., Morley D., Thompson M.A., Huang C., Abt G., Rothwell M., Cope E., Ntoumanis N. (2020). The effects of the iPlayClean education programme on doping attitudes and susceptibility to use banned substances among high-level adolescent athletes from the UK: A cluster-randomised controlled trial. Int. J. Drug Policy.

[52] Nilsson S., Allebeck P., Marklund B., Baigi A., Fridlund B. (2004). Evaluation of a health promotion programme to prevent the misuse of androgenic anabolic steroids among Swedish adolescents. Health Promot. Int..

[53] Ntoumanis N., Quested E., Patterson L., Kaffe S., Backhouse S.H., Pavlidis G., Whitaker L., Barkoukis V., Smith B.J., Staff H.R. (2020). An intervention to optimise coach-created motivational climates and reduce athlete willingness to dope (CoachMADE): A three-country cluster randomised controlled trial. Br. J. Sports Med..

[54] Ranby K.W., Aiken L.S., MacKinnon D.P., Elliot D.L., Moe E.L., McGinnis W., Goldberg L. (2009). A Mediation Analysis of the ATHENA Intervention for Female Athletes: Prevention of Athletic-Enhancing Substance Use and Unhealthy Weight Loss Behaviors. J. Pediatr. Psychol..

[55] Sagoe D., Holden G., Rise E.N.K., Torgersen T., Paulsen G., Krosshaug T., Lauritzen F., Pallesen S. (2016). Doping prevention through anti-doping education and practical strength training: The Hercules program. Perform. Enhanc. Health.

[56] Trenhaile J., Choi H.S., Proctor T.B., Work P. (1998). The effect of anabolic steroid education on knowledge and attitudes of at-risk preadolescents. J. Alcohol Drug Educ..

[57] Yager Z., McLean S.A., Li X. (2019). Body image outcomes in a replication of the ATLAS program in Australia. Psychol. Men Masc..

[58] Glasgow R.E., Linnan L.A. (2008). Evaluation of theory-based interventions. Health Behav..

[59] Sharma M. (2007). Behavioural interventions for preventing and treating obesity in adults. Obes. Rev..

[60] Darnton A. (2008). Reference Report: An Overview of Behaviour Change Models and Their Uses.

[61] Ajzen I. (1985). From Intentions to Actions: A Theory of Planned Behavior.

[62] Potter W.J. (2004). Theory of Media Literacy: A Cognitive Approach.

[63] Bandura A., McClelland D.C. (1977). Social Learning Theory.

[64] Janz N.K., Becker M.H. (1984). The Health Belief Model: A Decade Later. Health Educ. Q..

[65] Rubin A., Babbie E.R. (2017). Research Methods for Social Work.

[66] Goodstadt M.S. (1980). Drug education—A turn on or a turn off?. J. Drug Educ..

[67] Ringold D.J. (2002). Boomerang Effects in Response to Public Health Interventions: Some Unintended Consequences in the Alcoholic Beverage Market. J. Consum. Policy.

[68] Aidman E., Petróczi A., Hussain I., Deshmukh N., Nepusz T., Uvacsek M., Toth M., Barker J., Naughton D. (2010). Beyond self-report in doping: Validating declared substance use in sport with hair sample analysis. J. Sci. Med. Sport.

